# Intravitreal Therapy in Adults Aged ≤50 Years: Etiologic Spectrum, Treatment Patterns and Visual Outcomes in a Real-World Cohort

**DOI:** 10.3390/jcm15124508

**Published:** 2026-06-10

**Authors:** Carmen Antía Rodríguez-Fernández, David Oliver-Gutierrez, Albert Arnaiz, Tatiana Pablos, Gloria Segura-Duch, Miguel Ángel Zapata

**Affiliations:** 1 Ophthalmology Department, Ribera POVISA Hospital, 36211 Vigo, Spain; 2 FarmaChusLab Group, Health Research Institute of Santiago de Compostela (IDIS), 15706 Santiago de Compostela, Spain; 3 Ophthalmology Department, Verte Oftalmología Barcelona, 08006 Barcelona, Spain; davidoliguti@gmail.com (D.O.-G.); glo.segura.duch@gmail.com (G.S.-D.); 4 Department of Ophthalmology, Bellvitge University Hospital, 08907 Barcelona, Spain; 5 Ophthalmology Department, Hospital Universitari Vall d’Hebron, Universitat Autònoma de Barcelona, 08035 Barcelona, Spain; albert.arnaiz.7991@gmail.com (A.A.); tatianapablos96@gmail.com (T.P.); miguelangel.zapata@vallhebron.cat (M.Á.Z.)

**Keywords:** intravitreal injections, young adult, diabetes, macular edema

## Abstract

**Background:** Intravitreal injections (IVI) are widely used for the management of retinal diseases, yet younger adults are underrepresented in clinical trials and real-world reports. Data on the etiologic distribution and treatment patterns of IVI in patients aged ≤50 years remain limited. This study aimed to characterize the indications, treatment strategies, and visual outcomes of IVI in this age group. **Material and Methods:** Retrospective, single-center observational study including adults aged 18–50 years who received IVI between January 2020 and December 2023 at a tertiary referral hospital in Spain. Demographic data, diagnosis, treatment modality, regimen, number of injections and best-corrected visual acuity (BCVA) were collected. One eye per patient was included for analysis. Visual outcomes were assessed as change in logMAR BCVA between baseline and final follow-up. **Results:** A total of 122 patients were included. The most frequent indications were diabetic macular edema (29.5%), macular neovascularization of various etiologies (21.3%), retinal vein occlusion (13.9%) and uveitis (9.8%). Anti-vascular endothelial growth factor (anti-VEGF) agents were used in 80.3% of eyes, corticosteroids in 6.6% and combination therapy in 13.1%. The mean number of injections per patient was 6.0 ± 5.3 over a mean follow-up of 3.0 ± 2.5 years. Overall BCVA improved significantly from 0.50 ± 0.59 to 0.38 ± 0.50 logMAR (*p* = 0.012). **Conclusions:** IVI in adults ≤ 50 years is uncommon but encompasses a broad etiologic spectrum. Diabetic macular edema and macular neovascularization of diverse etiologies were the leading indications. Anti-VEGF therapy represented the main treatment modality in this cohort.

## 1. Introduction

Intravitreal injections (IVI) have revolutionized the management of retinal diseases over the past two decades. The introduction of anti-vascular endothelial growth factor (anti-VEGF) agents and long-acting corticosteroid implants has substantially improved the prognosis of conditions once considered blinding, such as neovascular age-related macular degeneration (AMD) [1], diabetic macular edema (DME) [2], retinal vein occlusion (RVO) [3], and uveitis-related macular edema [4].

Since the approval of anti-VEGF agents [5], the therapeutic landscape has expanded rapidly for AMD and other vascular diseases, enabling individualized treatment strategies ranging from fixed regimens to flexible pro re nata (PRN) and treat-and-extend (T&E) protocols. In parallel, intravitreal corticosteroid implants—such as dexamethasone (Ozurdex) and fluocinolone acetonide (Iluvien) [6]—have become established options for macular edema secondary to diabetes, RVO, or inflammation, but are also used for other off-label conditions [7,8].

Extensive research supports the efficacy and safety of these agents across multiple retinal pathologies. Most scientific output, however, originates from elderly populations, focusing on neovascular AMD [9], the leading cause of blindness in developed countries due to its high prevalence and socioeconomic burden. AMD rarely occurs before the age of 55 and is most common in individuals over 75 years. Consequently, younger adults (≤50 years) are underrepresented in clinical trials, and their therapeutic response and disease spectrum remain less well characterized.

Younger adults constitute a smaller but clinically heterogeneous group, in whom IVI indications include diabetic and thrombotic macular edema, inflammatory or myopic choroidal neovascularization, and other rare etiologies. Evidence on IVI outcomes in this population is scarce and largely limited to small retrospective studies in single-etiology analyses [10,11,12].

A clearer understanding of etiologic distribution, treatment patterns, and visual outcomes in younger adults is essential to refine therapeutic strategies and optimize long-term results. Identifying the most frequent indications and real-world effectiveness of IVI may help tailor regimens. Therefore, the present study aims to describe the etiologies, treatment patterns, and visual outcomes of intravitreal injections in adult patients aged 50 years or younger treated at a tertiary referral center.

## 2. Materials and Methods

### 2.1. Study Design and Participants

This was a retrospective, observational, single-center study conducted at the Department of Ophthalmology of a tertiary hospital in Spain. The study included adult patients aged 18–50 years who received IVI between January 2020 and December 2023. The protocol adhered to the tenets of the Declaration of Helsinki and was approved by the institutional Clinical Research Ethics Committee (CEIC) under reference code EOM(AG)004/2024(6230) (approval date: 1 March 2024). No identifiable patient information or images were used. Given the retrospective design and full data anonymization, the requirement for informed consent was waived by the CEIC.

Eligible participants were adults aged ≤50 years with any retinal disease requiring IVI. Inclusion criteria were intentionally broad to characterize real-world intravitreal therapy use across different retinal diseases in younger adults. Patients were included regardless of prior treatment status, disease chronicity, or previous therapies.

### 2.2. Clinical Assessment and Data Collection

Clinical data were retrieved from the hospital’s electronic medical records. For each patient, demographic information and ophthalmologic findings were collected, including age, sex, laterality/number of treated eyes, main indication for IVI, type of intravitreal drug, treatment regimen, number of injections, and baseline and final best-corrected visual acuity (BCVA). Clinical and treatment history were retrospectively reviewed from the first documented intravitreal injection available in the medical records through the end of follow-up. Final BCVA corresponded to the last available follow-up visit.

Diagnoses were confirmed through multimodal imaging—such as swept-source optical coherence tomography (SS-OCT), fluorescein and/or indocyanine green angiography, wide-field fundus photography, and fundus autofluorescence—when clinically indicated.

All patients received IVI with anti-VEGF agents (ranibizumab, aflibercept, brolucizumab, or bevacizumab), corticosteroid implants (dexamethasone or fluocinolone acetonide), or combination therapy. Anti-VEGF agent selection was based on disease indication and physician judgment according to routine clinical practice, and was not protocolized by the study design. Corticosteroid implants were administered per label or for selected off-label indications, such as uveitic or post-surgical macular edema. Loading doses, retreatment intervals, and treatment switching decisions were individualized according to disease characteristics, disease activity, response to treatment, and follow-up considerations within PRN or T&E treatment approaches.

### 2.3. Outcome Measures

The primary outcome was (1) the etiologic distribution of diseases requiring IVI, and the secondary outcomes were (2) treatment patterns (drug type, regimen, and number of injections) and (3) visual outcomes, expressed as the change in BCVA (logMAR) between baseline and final follow-up.

### 2.4. Statistical Analysis

Data were analyzed using Stata version 17 (StataCorp, College Station, TX, USA). When both eyes of the same patient were eligible, one eye was included for analysis to ensure data independence. When both eyes met inclusion criteria, one eye was randomly selected for analysis. This approach was used to minimize inter-eye correlation and preserve statistical independence.

Continuous variables were summarized as mean ± standard deviation (SD) or median with interquartile range (IQR), depending on normality. Categorical variables were expressed as absolute and relative frequencies. For comparisons, Chi-square or Fisher’s exact tests were used for categorical data, and Student’s *t*-test or the Mann–Whitney *U* test were used for continuous variables, as appropriate. Paired *t*-test or Wilcoxon signed-rank test was applied to assess visual changes before and after treatment. Kruskal–Wallis was used to compare visual changes among diagnostic groups. A *p*-value < 0.05 was considered statistically significant.

## 3. Results

**Figure 1:**
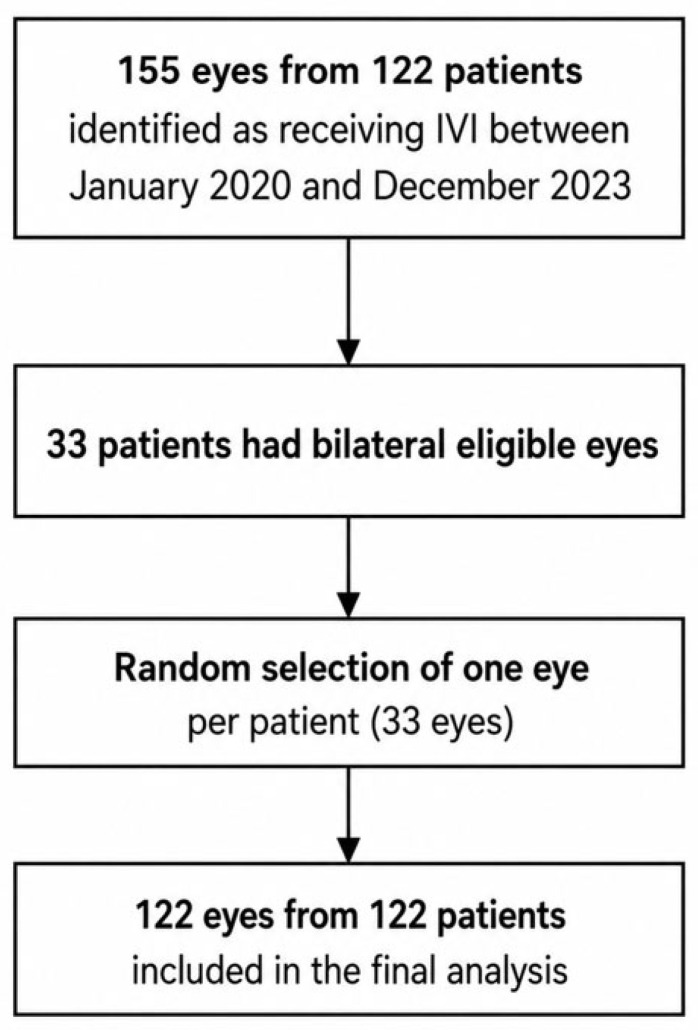
Flow diagram summarizing patient selection and eye inclusion. Abbreviations: IVI, intravitreal injection.

A total of 155 eyes from 122 patients were identified. Thirty-three patients had bilateral disease. To avoid inter-eye correlation, only one eye per patient was included in the analysis (*n* = 122) (Figure 1). The mean age was 40.3 ± 7.6 years (range 19–50) with a male predominance of 63.2%.

### 3.1. Etiologic Diagnosis

The most frequent diagnoses were diabetic macular edema (DME) (29.5%), followed by macular neovascularization (MNV) of multiple origins (21.3%), retinal vein occlusion (RVO) (13.9%), and uveitic causes (9.8%). All diagnoses are listed in Table 1.

### 3.2. Treatment Pattern

Type of intravitreal therapy: Anti-VEGF agents were administered in 80% of eyes, corticosteroids in 7%, and combined therapy in 13%. As multiple intravitreal agents could be used sequentially during follow-up, treatment categories were not mutually exclusive. Overall, 70% of patients received aflibercept, 50% ranibizumab, 11% bevacizumab, 2.5% brolucizumab, 18% dexamethasone implants, and 3.3% fluocinolone acetonide implants (Table 2). Among eyes with macular edema (diabetic or thrombotic), those receiving both anti-VEGF and corticosteroids received more injections (median 9, IQR 5–11) than those treated with anti-VEGF alone (median 4.5, IQR 1–10), although the difference did not reach statistical significance (*p* = 0.071, Mann–Whitney *U* test).

The mean number of intravitreal injections per patient was 6.0 ± 5.3 over a mean follow-up of 3.0 ± 2.5 years *(median 2.5 years, IQR 1.5–3.7)*.

Treatment regimen: Overall, the most common treatment regimen was PRN in 54.9% of patients, followed by T&E in 37.7%, and a single pre-surgical injection in 6.4%. The distribution of treatment regimens differed significantly across diagnostic groups (*p* = 0.001, Fisher’s exact test) (Table 3).

### 3.3. Visual Outcomes

Mean pre-treatment visual acuity was 0.50 ± 0.59 (95% CI 0.39 to 0.60) and improved significantly to 0.38 ± 0.50 (95% CI 0.29 to 0.48) after treatment (mean change = 0.11 ± 0.48; 95% CI 0.02–0.20; *p* = 0.012, Wilcoxon signed-rank test). When stratified by diagnosis, improvement was significant only for RVO (*p* = 0.01), but not for DME, uveitis or MNV. No significant differences were found in visual change among diagnostic groups (*p* = 0.49, Kruskal–Wallis test) (Table 4).

### 3.4. Safety

Medical records were reviewed for treatment-related complications. No clinically significant treatment-related adverse events, including endophthalmitis, retinal detachment, or other severe ocular complications, were identified during follow-up.

## 4. Discussion

This study provides a real-world characterization of intravitreal therapy in adults aged 50 years or younger, a subgroup rarely analyzed separately in retinal literature. Although intravitreal injections are predominantly performed in older populations, younger adults accounted for approximately 2% of the overall intravitreal treatment volume at our center during the study period. Despite their relatively low proportion, this group displayed considerable etiologic heterogeneity.

Diabetic macular edema (DME) was the leading indication. While large randomized trials have established the efficacy of anti-VEGF therapy in DME, younger patients are underrepresented in these studies, and age-specific real-world analyses are scarce. Consequently, direct comparisons with previously published series are not feasible. The predominance of DME in this cohort aligns with epidemiological evidence indicating earlier onset and longer disease duration of diabetes, which increases the risk of visually significant microvascular complications at younger ages [13]. Unlike neovascular age-related macular degeneration, which dominates older populations, metabolic retinal disease appears to be a major driver of IVI in working-age adults.

Age-restricted real-world analyses specifically addressing IVI in adults ≤ 50 years remain scarce. This is particularly relevant in the context of the increasing prevalence of diabetes in working-age populations and the growing impact of diabetes-related retinal disease in early adulthood, which remains a leading cause of visual impairment among working-aged individuals worldwide [14].

Macular neovascularization (MNV) was the second most frequent indication and included myopic, inflammatory and traumatic etiologies. Previous studies have shown that MNV in younger individuals is etiologically heterogeneous, with high myopia as a leading cause [15]. Although anti-VEGF therapy has demonstrated significant visual benefit in large studies of myopic MNV [16] and pachychoroid-related MNV [17], visual improvement in our cohort did not reach statistical significance. Several factors may explain this finding. First, baseline visual acuity was relatively preserved in many MNV cases, limiting the measurable gain (ceiling effect). Second, this group included highly heterogeneous entities with different pathogenic mechanisms and prognoses, including inflammatory, traumatic and idiopathic etiologies. Finally, the relatively small sample size may have limited statistical power.

Retinal vein occlusion (RVO) accounted for 13.9% of cases and was the only subgroup demonstrating statistically significant visual improvement. Age-related differences in treatment response have been described, with younger patients often showing greater functional recovery following anti-VEGF therapy [18]. In our cohort, the larger visual gain in RVO compared with chronic entities such as DME may reflect a more acute onset and less irreversible structural damage at baseline.

Anti-VEGF agents represented the main therapeutic approach across most diagnostic groups. Corticosteroid implants were primarily used in uveitic macular edema and selected refractory cases across other diagnostic groups. Their effectiveness in younger patients with inflammatory or vascular macular edema has been previously reported [11,12]. In our cohort, eyes receiving combination therapy required a higher number of injections compared with anti-VEGF monotherapy. This likely reflects greater disease chronicity or incomplete response rather than reduced treatment efficacy. Sequential use of different drug classes inherently increases cumulative injection numbers and is typically reserved for more complex cases. The off-label use of corticosteroid implants for selected non-diabetic etiologies, such as Irvine–Gass syndrome or radiation maculopathy, has also been described in the literature [7,8], supporting their role when inflammation contributes significantly to the disease process.

Overall visual improvement in the cohort was modest. Interpretation of pooled visual outcomes should be considered in the context of heterogeneous indications and treatment objectives across diagnostic groups. In particular, the heterogeneous nature of the “Others” category should be considered, as some indications, including pre-surgical IVI, had therapeutic goals not primarily focused on visual improvement. Relatively preserved baseline visual acuity in several diagnostic groups, particularly DME, may have limited measurable functional gain (ceiling effect). Additionally, chronic microvascular or inflammatory damage may constrain visual recovery despite anatomical control of edema. In contrast, macular edema secondary to RVO typically presents as an acute event with poorer initial visual acuity but relatively preserved retinal architecture, providing a wider margin for functional recovery once vascular permeability is reduced. These pathophysiological differences may explain why visual improvement reached statistical significance in RVO but not in DME or other chronic etiologies, despite comparable IVI strategies.

The heterogeneity in treatment regimens observed—particularly the coexistence of PRN and T&E—reflects the need for individualized approaches in this population. The predominance of a PRN regimen in our cohort may reflect the generally lower injection burden observed in younger patients with non-AMD retinal diseases. Registry data from the IRIS database in pediatric populations indicate that most young patients require three or fewer anti-VEGF injections to achieve MNV stabilization [19], contrasting with the chronic retreatment patterns seen in neovascular AMD. Similarly, in central RVO, younger individuals have been shown to require significantly fewer intravitreal injections to achieve resolution compared with patients older than 50 years [20]. Although our cohort included adults rather than children, the absence of degenerative AMD and the predominance of secondary, inflammatory or vascular etiologies may partly explain the preference for reactive PRN strategies and the relatively moderate injection burden observed.

The safety profile observed in this study was consistent with previously reported data, with no severe treatment-related complications recorded during follow-up, although minor adverse events may have been underreported because of the retrospective design.

Unlike older cohorts dominated by age-related macular degeneration MNV, the etiologic spectrum in younger adults reflects a higher proportion of metabolic, inflammatory and myopic mechanisms. Younger adults may also differ from elderly populations in disease chronicity and systemic comorbidity burden, factors that may influence treatment needs and visual outcomes. Registry data and age-restricted series indicate that younger patients often present with better baseline visual acuity, require fewer intravitreal injections, and may achieve more favorable short-term functional outcomes compared with elderly populations [11,12,19]. Our findings align with this literature.

In our cohort, the predominance of DME underscores the relevance of metabolic retinal disease in this age group. The requirement for IVI likely reflects cumulative exposure to chronic metabolic dysregulation and highlights the importance of timely ophthalmologic management to prevent irreversible structural damage.

The main limitations of this study include its retrospective design (follow-up heterogeneity, non-standardized treatment allocation), single-center setting, and the inherent heterogeneity of diagnoses within this under-50 population. Some diagnostic subgroups included relatively small sample sizes; therefore, subgroup analyses should be considered exploratory and interpreted cautiously. In addition, formal correction for multiple comparisons was not performed because these analyses were secondary to the primary descriptive and epidemiologic objectives of the study.

As this study was conducted at a tertiary referral center, some degree of selection bias toward more complex or refractory cases may have influenced the observed etiologic spectrum and treatment patterns, potentially limiting generalizability to other ophthalmologic settings. Furthermore, systemic treatments, inflammation control, lens status changes, and minor adverse events were not systematically evaluated and may have influenced visual outcomes.

Despite these limitations, the study provides a structured real-world overview of intravitreal therapy patterns in a population that remains underrepresented in retinal literature. Future multicenter prospective studies may help better characterize disease-specific responses and long-term safety outcomes in younger adults.

## 5. Conclusions

Intravitreal therapy in adults aged ≤50 years represents a small proportion of overall injection volume but encompasses a broad and distinct etiologic spectrum compared with older populations. DME and MNV represented the leading indications, while inflammatory and vascular conditions also contributed significantly.

Anti-VEGF agents remain the primary treatment modality in this cohort, with corticosteroid implants used in selected inflammatory or refractory cases.

These findings provide baseline real-world data on intravitreal treatment patterns and outcomes in younger adults and may serve as a reference for future age-comparative studies.

## Figures and Tables

**Table 1:** Diagnostic groups used in the analyses and specific diagnoses included within each category.

Diagnostic Group	*n* (%)	Specific Diagnosis	*n* (%)
DME	36 (29.5%)	Diabetic macular edema	36 (29.5%)
RVO	17 (13.9%)	Retinal vein occlusion with macular edema	15 (12.3%)
		Retinal vein occlusion without macular edema	2 (1.6%)
Uveitis	12 (9.8%)	Intraocular inflammation	8 (6.6%)
		Uveitic macular edema	4 (3.3%)
MNV	26 (21.3%)	Myopic MNV	10 (8.2%)
		Uveitic MNV	5 (4.1%)
		MNV secondary to choroidal rupture	3 (2.5%)
		Idiopathic MNV	1 (0.8%)
		Optic nerve drusen-associated MNV	1 (0.8%)
		Best dystrophy-associated MNV	1 (0.8%)
		Serpiginous chorioretinopathy-associated MNV	1 (0.8%)
		Polypoidal MNV	1 (0.8%)
		Pachychoroid-associated MNV	3 (2.5%)
Others	31 (25.4%)	Neovascular glaucoma	2 (1.6%)
		Proliferative diabetic retinopathy (pre-surgical IVI)	7 (5.7%)
		Intraocular tumors (melanoma, osteoma, vasoproliferative tumor, and hemangioma)	5 (4.1%)
		Radiation retinopathy	1 (0.8%)
		MacTel type 1 or Coats disease	4 (3.3%)
		Central serous chorioretinopathy without MNV	5 (4.1%)
		Irvine–Gass syndrome	2 (1.6%)
		Unclassified ischemic retinopathy	1 (0.8%)
		Traumatic macular hemorrhage	1 (0.8%)
		Dome-shaped maculopathy with subretinal fluid without evidence of MNV	2 (1.6%)
		Retinal neovascularization secondary to occlusive vasculitis	1 (0.8%)
Total	122 (100%)	Total	122 (100%)

*n* indicates the number of eyes, and percentages refer to the total sample (*n* = 122). Each eye was assigned to a single diagnostic group based on the primary diagnosis used for the analyses. Abbreviations: DME, diabetic macular edema; RVO, retinal vein occlusion; MNV, macular neovascularization; MacTel, macular telangiectasia.

**Table 2:** Type of intravitreal therapy, injection burden, and follow-up duration by diagnostic group.

Diagnosis	Anti-VEGF	Corticosteroids	Both	Number of IVI	Mean Follow-Up, Years
DME	31 (86%)	0	5 (13.9%)	6.8 (6.2)	3.2 (2.1)
RVO	14 (82.4%)	0	3 (17.7%)	7.8 (5.7)	2.8 (1.9)
Uveitis	2 (16.7%)	8 (66.7%)	2 (16.7%)	3.3 (3.1)	2.6 (1.7)
MNV	23 (88.5%)	0	3 (11.5%)	6.6 (4.1)	3.7 (3.4)
Others	28 (90.3%)	0	3 (9.7%)	4.7 (5.2)	2.7 (2.6)
Total	98 (80.3%)	8 (6.6%)	16 (13.1%)	6.0 (5.3)	3.0 (2.5)

Anti-VEGF therapy includes aflibercept (Eylea), ranibizumab (Lucentis), bevacizumab (Avastin), and brolucizumab (Beovu). Corticosteroid therapy includes dexamethasone implant (Ozurdex) and fluocinolone acetonide implant (Iluvien). The combined category refers to eyes that received both anti-VEGF and corticosteroid treatments at any time during follow-up. Time represents years. Data in () refers to percentages or standard deviations. No biosimilar formulations were used during the study period. Abbreviations: IVI, intravitreal injections; DME, diabetic macular edema; RVO, retinal vein occlusion; MNV, macular neovascularization.

**Table 3:** Treatment regimen by diagnostic groups.

Diagnosis	T&E	PRN	Single Pre-Surgical	Total
DME	14 (38.9%)	22 (62.86%)	-	36
RVO	10 (58.8%)	7 (41.2%)	-	17
Uveitis	8 (66.7%)	4 (33.3%)	-	12
MNV	6 (23.1%)	20 (76.9%)	-	26
Others	8 (25.8%)	14 (45.2%)	9 (30.0%)	31
Total	46 (37.7%)	67 (54.9%)	9 (6.4%)	122

T&E (treat-and-extend) refers to a proactive regimen with progressively extended intervals between injections based on disease activity. PRN (pro re nata) refers to reactive retreatment based on predefined recurrence criteria. Abbreviations: DME, diabetic macular edema; RVO, retinal vein occlusion; MNV, macular neovascularization.

**Table 4:** Change in best-corrected visual acuity (logMAR) by diagnosis.

Diagnosis	N	Baseline BCVA	SD	BCVA Change *	SD	p (Pre vs. Post, Wilcoxon)	K-Wallis
DME	36	0.41	0.56	−0.07	0.43	0.21	*p* = 0.49
RVO	16	0.69	0.74	−0.28	0.50	0.01	
Uveitis	12	0.275	0.21	−0.13	0.27	0.11	
MNV	26	0.37	0.45	−0.10	0.37	0.25	
Others	32	0.70	0.67	−0.06	0.64	0.98	
Total	122	0.50	0.59	−0.11	0.48	0.012	

* BCVA = best-corrected visual acuity. Negative values of BCVA change indicate visual improvement (lower logMAR values after treatment). *p*-values correspond to within-group comparisons using the Wilcoxon signed-rank test and between-group comparisons using the Kruskal–Wallis test. Only one randomly selected eye per patient was included when both eyes were eligible. Abbreviations: DME, diabetic macular edema; RVO, retinal vein occlusion; MNV, macular neovascularization.

## Data Availability

Data supporting the findings of this study are available from the corresponding author upon reasonable request.
